# Medical students as primary care providers: a novel curriculum enhancing understanding of chronic disease management

**DOI:** 10.5116/ijme.5787.683e

**Published:** 2016-08-13

**Authors:** Rebecca Liu, Kevin W. Su, Serene I. Chen, Pinar Oray-Schrom

**Affiliations:** 1 Department of Internal Medicine, Yale School of Medicine, USA

**Keywords:** Medical students, primary care, novel curriculum, chronic disease management

## Introduction

The traditional model of medical education has been criticized for providing a fragmented learning experience that fails to engage students in patient-centered care.^1^^-^^3^ Because students rotate between specialties each month of their clinical year, they develop only brief therapeutic relationships with individual patients, rarely assume ownership of patient care, and are unable to contribute to chronic disease management or appreciate the long-term effects of medical interventions. We thus present a newly structured clerkship in which medical students, overseen by preceptors, function as long-term primary care providers (PCPs) for a personal panel of patients. Moreover, because the clerkship is integrated into the operation of our institution’s Primary Care Center (PCC), our model has several unique features that facilitate implementation of medical student education in the outpatient setting and the delivery of high-quality patient care.

### Clinic mission

Wednesday Evening Clinic (WEC) is a student-staffed clinic recently integrated into the PCC of the Yale-New Haven Hospital (YNHH). It was established with the mission of providing an extensive primary care experience for senior Yale medical students, in which they manage their own cohort of approximately 30 adult patients as PCPs under supervision of attending physicians. Each year, up to 15 Yale medical students beyond their third year are selected to participate in WEC weekly for one year or more. Medical students take ownership of multiple aspects of patients’ care, including general health maintenance, chronic disease management, age-appropriate screening, counseling, and patient-care coordination. WEC primarily serves an underserved patient population mostly insured by Medicaid and Medicare.

### Clinic operations

Clinic night starts with a 30-minute student-led teaching conference covering important topics in primary care. Teaching is done in the format of patient case presentations and interactive group discussions with topics and questions selected from the Yale Office-Based Medicine Curriculum, which is a comprehensive primary care curriculum used by more than 170 Internal Medicine and Primary Care Residency programs.

Each medical student provider is responsible for seeing three patients per clinic night. Students are expected to review patients’ medical history documented in the electronic medical record prior to their encounters. During a clinic visit, each student independently takes a history and performs a physical examination, then formally presents to an attending physician.  After discussing the assessment and plan, the student and attending physician return to the patient to repeat pertinent parts of the history and physical examination, and to discuss the plan with the patient. Every encounter is documented by the student in the patient’s electronic medical record and then addended and co-signed by the supervising attending physician. In addition to providing care on clinic nights, students offer follow-up care (e.g. discuss test results, address questions and medication refill requests, and coordinate social services) for their patients throughout the week.

Clinic structure is modeled after standard resident clinics, with student providers divided into three patient care teams, each led by a designated team-attending physician. This team structure ensures that each student is supervised by one designated faculty team-attending physician throughout the year. In addition to precepting students on Wednesday nights, team-attendings are also the MDs of record for the patient cohort, responsible for overseeing the medical care of these patients. Team-attendings also supervise follow-up care performed by students in between patient encounters. Students receive one-on-one evaluation at least 3 times per year from team-attending physicians. Each evaluation session includes being observed for an entire clinic visit, completing a Mini-Clinical Evaluation Exercise (Mini-CEX), followed by formal feedback sessions. On clinic nights, a pool of voluntary attending physicians from the Greater New Haven area and third-year Yale Internal Medicine residents also participate in the clinic on a rotating basis. Their role consists of observing and teaching the medical students and supervising patient care.

### The unique features of WEC

To the best of our knowledge, this medical student clinic is unique in many ways. First, WEC provides comprehensive primary care for their patients over multiple years, unlike most student-run clinics, which focus on screening or providing acute and bridging care.^4^ Furthermore, WEC students participate in clinic for one year or more, and are required to attend a minimum of 36 clinic sessions each year, in contrast to the sporadic and unpredictable provider attendance that is common to most student-run clinics. This year-long commitment ensures a sense of ownership, and patients recognize and identify students as their PCPs. Second, WEC is fully integrated into the YNHH PCC, which allows access to fully equipped examination rooms, charting rooms, nursing stations, and a clinical laboratory. WEC also operates with full ancillary services staffed by hospital employees (e.g. nursing, laboratory, social work, scheduling, and referral infrastructure). This structural integration allows students to experience the dynamics of patient care within a large health care system, and also benefits patients who prefer evening hours that are otherwise not available at the PCC (WEC is open to all patients of the YNHH PCC). Third, WEC attendings consist of 3 full-time faculty team-attendings and a pool of rotating volunteer MDs from YNHH and the Greater New Haven community. The three team-attendings are selected from faculty members of the General Internal Medicine section, and are experienced clinician-educators who routinely supervise and mentor residents in the PCC during the daytime. They are also able to offer patients the same level of care in the student clinic as the regular PCC. Fourth, all 3rd year Yale Internal Medicine residents regularly attend the clinic as part of their ambulatory curriculum requirement and assist with observing, teaching and evaluating medical students. Finally, WEC maintains its own panel of patients and is financially sustained through billed encounter revenues and through the support from the School of Medicine and YNHH.

### Educational experience

A questionnaire survey of WEC alumni indicates that participation in WEC enhanced students’ overall comfort in providing outpatient care. Furthermore, students deemed WEC valuable for their educational experience because of features that are otherwise not found in traditional clinical clerkships, including unmatched autonomy and level of responsibility, ability to maintain long-term physician-patient relationships, and opportunity to observe the development of chronic diseases and the effectiveness of management plans over time.

## Conclusions

WEC offers a novel combination of increased student responsibility and continuity of care rarely encountered in the traditional medical school curriculum. Moreover, our model contains elements that are adaptable for other institutions. Our model’s structural features (integration into an existing clinic, ancillary staff support, and the ability to generate some revenue) facilitate implementation and create an environment capable of providing both high-quality training and patient care. Future studies should describe and compare educational outcomes (both objective and subjective) experienced by students enrolled in WEC versus students participating in traditional ambulatory rotations. The experiences of preceptors and patients participating in WEC also remain areas for further investigation. Further development and implementation of ambulatory experiences emphasizing chronic disease management and continuity of care into medical school curricula are warranted.

### Conflicts of Interest

The authors declare that they have no conflict of interest.
